# Longitudinal five-year follow-up by muscle MRI and high-resolution nerve ultrasound in hereditary neuropathies

**DOI:** 10.1186/s42466-026-00508-1

**Published:** 2026-06-23

**Authors:** Kim L. Vockert, Natalie Winter, Friederike S. Kirschmann, Jörg B. Schulz, Christiane Kuhl, Sven Nebelung, Teresa Lemainque, Maike F. Dohrn

**Affiliations:** 1 Department of Neurology, Medical Faculty, RWTH Aachen University, Pauwelsstr. 30, Aachen, 52074 Germany; 2 Department of Neurology and Hertie Institute for Clinical Brain Research (HIH), University Hospital Tübingen, Tübingen, Germany; 3 Department of Child and Adolescent Psychiatry, Psychosomatics and Psychotherapy, Medical Faculty, RWTH Aachen University, Aachen, Germany; 4 Department of Diagnostic and Interventional Radiology, Medical Faculty, RWTH Aachen University, Aachen, Germany

**Keywords:** Biomarker, Fat fraction, Muscle echogenicity, Ultrasound pattern sum score, Charcot-Marie-Tooth disease

## Abstract

**Background and Aims:**

Hereditary polyneuropathies are rare heterogeneous neurological disorders that cause progressive muscle weakness and disability. As new therapies emerge, sensitive imaging biomarkers to monitor disease progression are essential. This exploratory longitudinal study evaluated MRI with focus on the lower limbs and high-resolution ultrasound (HRUS) at two points over the period of 4.75 years in genetically confirmed patients, and compared imaging with clinical and electrophysiological findings.

**Methods:**

Fourteen patients (CMT1A, CMT1X, CMT1B, CMT1C, CMT2, and hereditary myoneuropathy) underwent standardized clinical examinations, including Charcot-Marie-Tooth Examination Score version 2 (CMTESv2), Medical Research Counsil muscle strength, and sensory testing. Nerve conduction studies (NCS) were performed on upper limb nerves. MRI of lower limbs evaluated muscle volume, normalized T1w signal intensity (nSI), and proton density fat fraction (PDFF). HRUS was used to assess nerve cross-sectional areas, the ultrasound pattern sum score (UPSS), and muscle echogenicity.

**Results:**

Over the observation interval, patients showed a significant increase in CMTESv2 (*p* = 0.0031), especially in motor symptoms (*p* = 0.0012). MRI-derived muscle volumes did not change; however, nSI significantly increased in gastrocnemius lateralis muscles (*p* = 0.0023), indicating progressive fat infiltration. UPSS increased in demyelinating polyneuropathies (*p* = 0.0299) and was inversely correlated with nerve conduction velocities of the median (*p* = 0.0413) and ulnar nerves (*p* = 0.0064).

**Interpretation:**

Over this 5-year interval, our cohort showed clinical disease progression, which was not reflected by NCS and MRI volumetry. In contrast, MRI-derived nSI correlated with PDFF and captured progressive muscle fat replacement, correlating with declining muscle strength. UPSS showed longitudinal progression in demyelinating neuropathies. PDFF and UPSS are promising biomarkers and may support outcome evaluation in future clinical trials.

## Introduction

Hereditary polyneuropathies are rare neurological disorders with distal muscle atrophy and weakness being key symptoms [1–4]. With new therapeutic options for Charcot-Marie-Tooth disease (CMT) under investigation [5,6], it becomes even more important to define objective outcome parameters for trials and clinical practice. Due to the rarity of each specific disease subtype and the lack of longitudinal studies, it has been challenging to date to establish standardized biomarkers for disease progression and treatment response.

MRI enables comprehensive assessment of structural changes in muscles and surrounding tissues, providing quantitative data on muscle volume and fat fraction [7].

Previous evaluation of semi-automated MRI muscle volumetry showed that hereditary and acquired polyneuropathies differed considerably in distal leg muscle volumes [8]. Other works have shown that in hereditary polyneuropathies, muscle fat fraction correlated with clinical severity and electrophysiological measures [9–11]. The few longitudinal studies in this field show progression in fat fraction [12, 13]. For instance, Evans et al. (2025) demonstrated a consistent progression of the proton-density fat fraction (PDFF), an MRI-derived biomarker, in the calf muscles of CMT1A patients over four years, as well as a significant correlation with the CMT examination score.

High-resolution ultrasound (HRUS) is a promising, non-invasive tool for characterizing peripheral nerve structures and superficial muscle groups [14]. In a previous study, we have shown that muscle echogenicity correlates inversely with Medical Research Counsil (MRC) muscle strength and that nerve cross-sectional area (CSA) measurements can help distinguish between demyelinating and axonal neuropathies [15]. Others have already suggested combining MRI and nerve ultrasound to identify affected nerve segments for the diagnosis of peripheral neuropathies [16]. Only a few published studies compare MRI and ultrasound findings in muscles [17]. The study by Mul et al. (2018) showed a strong correlation between echo intensity in ultrasound imaging and fat fraction in MRI in patients with facioscapulohumeral muscular dystrophy. To the best of our knowledge, no studies have specifically investigated this correlation in cohorts of hereditary polyneuropathy.

Our study‘s primary objective was to evaluate the sensitivity of muscle MRI and HRUS for detecting longitudinal disease progression in patients with hereditary polyneuropathies. Specifically, we aimed to determine whether MRI-derived and HRUS-derived parameters reflect clinical progression over a follow-up period of approximately five years. We addressed three main issues: (i) do MRI-derived parameters and HRUS-derived parameters demonstrate progressive muscle fat infiltration and correlate with clinical measures of weakness? (ii) Is muscle volumetry sensitive to longitudinal changes? (iii) Does the UPSS effectively capture disease progression, particularly in demyelinating neuropathies?

The present study analyzes a cohort derived from routine clinical care, reflecting everyday clinical practice and aims for an exploratory approach. Our approach integrated MRI and HRUS. By correlating these imaging findings with detailed clinical and electrophysiological assessments, we aimed to assess the strengths and limitations of these imaging techniques for evaluating longitudinal changes in hereditary polyneuropathies.

## Methods and materials

### Patient selection

Patients were recruited between June 2018 and September 2019 at the Neuromuscular Outpatient Clinic, Department of Neurology, RWTH Aachen University Hospital, Aachen, Germany. We obtained written informed consent before study enrollment. The study was conducted in accordance with the Declaration of Helsinki and was approved by the local institutional review board (EK215-18, CTC-A22-338). Inclusion criteria were adulthood (≥ 18 years), a genetically confirmed diagnosis of hereditary neuropathy, and no contraindications for MRI, such as cardiac pacemakers or defibrillators not certified as MRI-safe, or any other non-MRI-compatible implants or devices, e.g., ferromagnetic cerebral clips or cochlear implants. Initially, we recruited 20 patients. During the follow-up period, one patient died. Three patients did not participate in the follow-up examination and were excluded. 15 patients underwent clinical evaluation, whole-body MRI, and high-resolution ultrasound prospectively at two time points: baseline and follow-up. Follow-up visits were scheduled 4 to 5 years after the baseline visit. All patients were examined by the same four trained examiners (KLV, FSK, NW, MFD). One patient was excluded from the final analysis because they were the only participant to receive disease modifying treatment for ATTRv-amyloidosis so that the study would not have measured natural history of disease.

### Clinical examination

A detailed medical history was taken for all patients, including ongoing symptoms such as numbness, tingling, cramps, hyperesthesia, hypoesthesia, neuropathic pain, gait instability, disturbance of fine motor skills, and autonomic complaints (e.g. abnormal sweating, bowel movement abnormalities). Clinical examination was performed for all patients. The muscle strength of the upper and lower limbs was determined using the MRC muscle scale [18], ranging from 0 to 5, where 0 indicates no visible contraction and 5 indicates normal strength. We calculated the MRC sum score adding together the values of the deltoid and biceps brachii muscles, wrist extension, iliopsoas, quadriceps femoris and tibial anterior muscles, and toe elevation muscles on both sides. The MRC sum score ranges from 0 (total paralysis) to 70 (full muscle function) points. In addition, we analyzed the gait pattern, specifically looking for steppage gait or afferent ataxia.

We further inspected the undressed extremities for distal and proximal muscle asymmetries and atrophy, and musculoskeletal abnormalities such as pes cavus and claw toes. Tendon reflex levels were examined at the triceps, biceps, Achilles, and patellar tendons. Our sensory examination included the perception and levels of abnormality for touch, cold, pinprick, vibration, and position perception in the upper and lower limbs.

To measure overall disease severity, we used the *Charcot-Marie-Tooth Examination Score version 2* (CMTESv2) [19, 20], a scale ranging from 0 to 28 points, with higher scores indicating greater severity.

### Nerve conduction studies

Nerve conduction studies (NCS) were performed on the non-dominant arm of all patients using the same neurophysiology device (Natus Neurology, Nicolet EDX). Two motor nerves (the ulnar nerve and the median nerve) and one sensory nerve (the radial nerve) were measured as part of the CMTNS score by default. For the motor nerves, we assessed distal motor latencies (DML), compound motor action potential amplitudes (CMAP), nerve conduction velocities (NCV), and F-wave latencies. For the sensory nerve, we measured the nerve conduction velocity and the sensory action potential (SNAP).

### MRI studies and image analysis

All patients underwent whole-body muscle MRI at the Department of Diagnostic and Interventional Radiology, RWTH Aachen University Hospital, Aachen, Germany. The MRI examinations were performed on three clinical 1.5T MRI scanners, Philips Achieva, Philips Ingenia, or Philips Ingenia Ambition X (Philips Healthcare, Best, The Netherlands), using the integrated volume transmitter/quadrature detection receiver coil (Q body coil) for the Achieva scanner and the dStreamWholeBody coil setup for the Ingenia scanners. The imaging protocol consisted of axial T1-weighted (T1w) turbo spin echo (TSE), axial 2D short tau inversion recovery (STIR), and axial 3D multi-echo gradient echo mDIXON Quant sequences for chemical shift-based determination of the proton density fat fraction (PDFF). At the time of the first examination, T1w TSE and STIR sequences were performed on the patients, covering head to toe by multiple consecutive stacks of slices.

All MRI sequence parameters are shown in Table 1. For mDIXON Quant, we selected a short echo spacing of 0.8 ms to minimize concomitant gradient field-induced phase errors [21]. Water and fat images as well as PDFF, T2* and R2* maps were reconstructed on the MRI workstation using the scanner software.

**Table 1:** MRI sequence parameters

Parameter (scanner)	T1w TSE (Achieva)	T1w TSE (Ingenia)	STIR (Achieva)	STIR (Ingenia)	mDixon quant (Ingenia)
Technique	Spin echo	Spin echo	Spin echo	Spin echo	Gradient echo
TR [ms]	902	620	3972	2147	6.4
TE [ms]	17	9	64	60	1.07 + n*0.8 with n = {0,1,2,3,4,5}
TSE Factor	6	7	30	17	**N/A**
Flip angle	90°	90°	90°	90°	5°
FOV [mm]	529 × 529	542 × 542	500 × 500	500 × 500	500 × 500
Acquisition matrix [pixel]	436 × 348	399 × 308	336 × 252	384 × 297	256 × 256
Acquired pixel size [mm/pixel]	1.2 × 1.5	1.4 × 1.8	1.5 × 2.0	1.3 × 1.7	1.95 × 1.35
Reconstruction matrix [pixel]	448 × 448	512 × 512	512 × 512	448 × 448	480 × 480
Reconstruction pixel size [mm/pixel]	1.2 × 1.2	1.1 × 1.1	1.0 × 1.0	1.1 × 1.1	1.0 × 1.0
Slice thickness [mm]	6	5	6	5	4
Slice gap [mm]	7	6.2	7	6.2	4
NSA	2	1	2	1	2

T1w = T1-weighted; TSE = turbo spin echo; STIR = short tau inversion recovery; TR = repetition time; TE = echo time; FOV = field of view; NSA = number of signal averages; N/A = not applicable

**Fig. 1:**
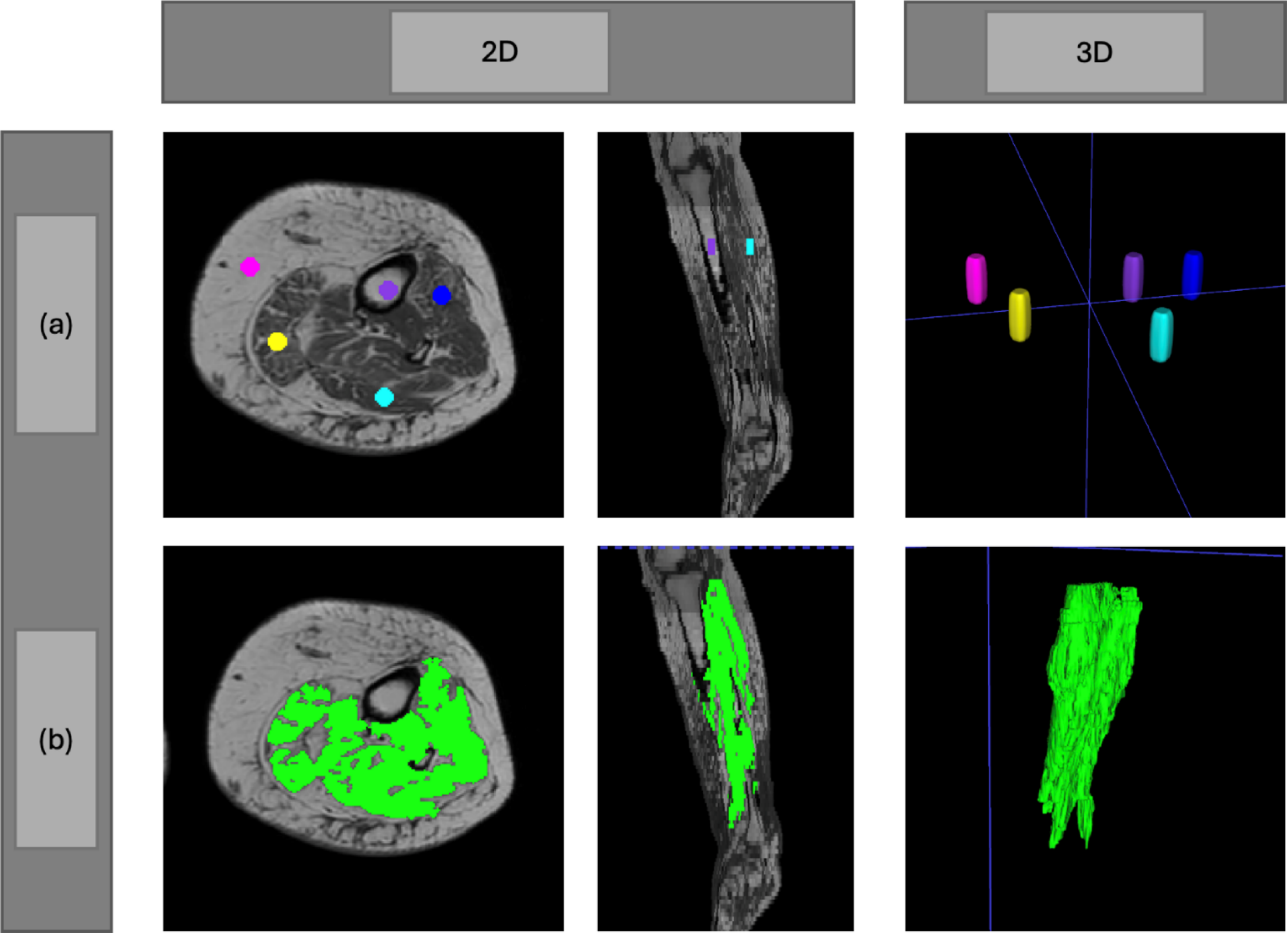
Semi-automated muscle segmentation and region-of-interest (ROI) placement in lower leg MRI. Example of image analysis in a patient with hereditary neuropathy (CMT1C). (a) ROI placement (colored circles) in muscle, fat, and bone marrow on an axial T1-weighted image (left), with the corresponding sagittal reconstruction (center) and 3D representation of the ROI positions (right). (b) Semi-automated segmentation of lower leg musculature (green overlay) performed with ITK-SNAP on axial slices (left), sagittal reconstructions (center), and 3D renderings (right). These approaches were used to quantify muscle volume, normalized T1 signal intensity (nSI), and proton density fat fraction (PDFF)

Pseudonymized data were converted from DICOM to NIFTI for further processing. For volumetry, T1w-MRI datasets were semi-automatically segmented using ITK-SNAP [22–24], an open-source segmentation software (version 4.0.2, https://www.itksnap.org/*)*, measuring muscle volumes in the patient’s left upper and lower leg (Fig. 1a). We used the active contour algorithm of ITK-SNAP for automated segmentation, followed by manual refinement. The segmentations were performed by FSK at baseline and by KLV, who was trained by FSK, at follow-up. All segmentations were validated by MFD. The region of muscle segmentation for the upper leg was defined from the femoral head to the base of the patella. For the lower leg, the defined region extended from the medial tibial condyle to the inferior articular surface of the tibia. The need for manual correction varied from patient to patient and depended mainly on the degree of muscle atrophy. In the case of severe muscle atrophy, the automatic segmentation needed more improvement.

For further analysis, cylindrical *regions of interest* (ROI) were placed in muscle, fat, and bone marrow tissues of the lower leg to determine mean T1w signal intensity (SI) and mean PDFF for each ROI (Fig. 1b). The muscles differentiated here included proximal muscles (rectus femoris (RF) and vastus lateralis (VL) muscle) and distal muscles (tibialis anterior (TA), lateral and medial gastrocnemius (GCNL and GCNM) muscles). Proximal muscles were measured just underneath the minor trochanter of the femur, whereas distal muscles were measured at the level of the tibial tuberosity. For each patient, a fixed brush size of 6 to 10 pixels in diameter was selected based on the patient’s anatomical characteristics to cover as much muscle tissue as possible. For each individual patient, the brush size was kept constant at baseline and follow-up. The ROIs extended over four axial layers in the T1w images and six axial layers in the PDFF images, covering the same volume while accounting for the different slice thicknesses. We normalized all muscle mean SIs to the tibial or femoral bone marrow mean SI resulting in a normalized SI (nSI) to facilitate comparison between patients. PDFF was only measured at follow-up. As this precluded a direct longitudinal comparison of PDFF, we correlated PDFF values with nSI, revealing a strong correlation (*p* < 0.0001, R² = 0.9). Based on this correlation, we determined the calibration curve. This method was used to validate nSI as a surrogate marker for PDFF within our cohort.

### Nerve and muscle ultrasound

High-resolution ultrasound (HRUS) for systematic muscle and nerve sonography was performed using a high-frequency broadband linear array 14 MHz probe, Mindray TE7 (Mindray Medical Germany GmbH, Darmstadt, Germany). NW and MFD performed the ultrasound examination at baseline and follow-up. The patients were examined according to the ultrasound pattern sum scores (UPSS) protocol [25]. For this purpose, the nerve cross-sectional area (CSA) of the median nerve, ulnar nerve, superficial radial nerve, tibial nerve, common peroneal nerve, superficial peroneal nerve, sural nerve, and vagus nerve was measured at fixed points. CSA was measured using the trace function without allowing zoom magnification [26, 27]. The diameter of the C5 and C6 nerve roots was measured at the exit point of the foramina. The UPSS was determined from the pattern of nerve enlargement. It ranged from zero, when no nerve enlargement was present, to 22, when all nerves were enlarged [25].

**Fig. 2:**
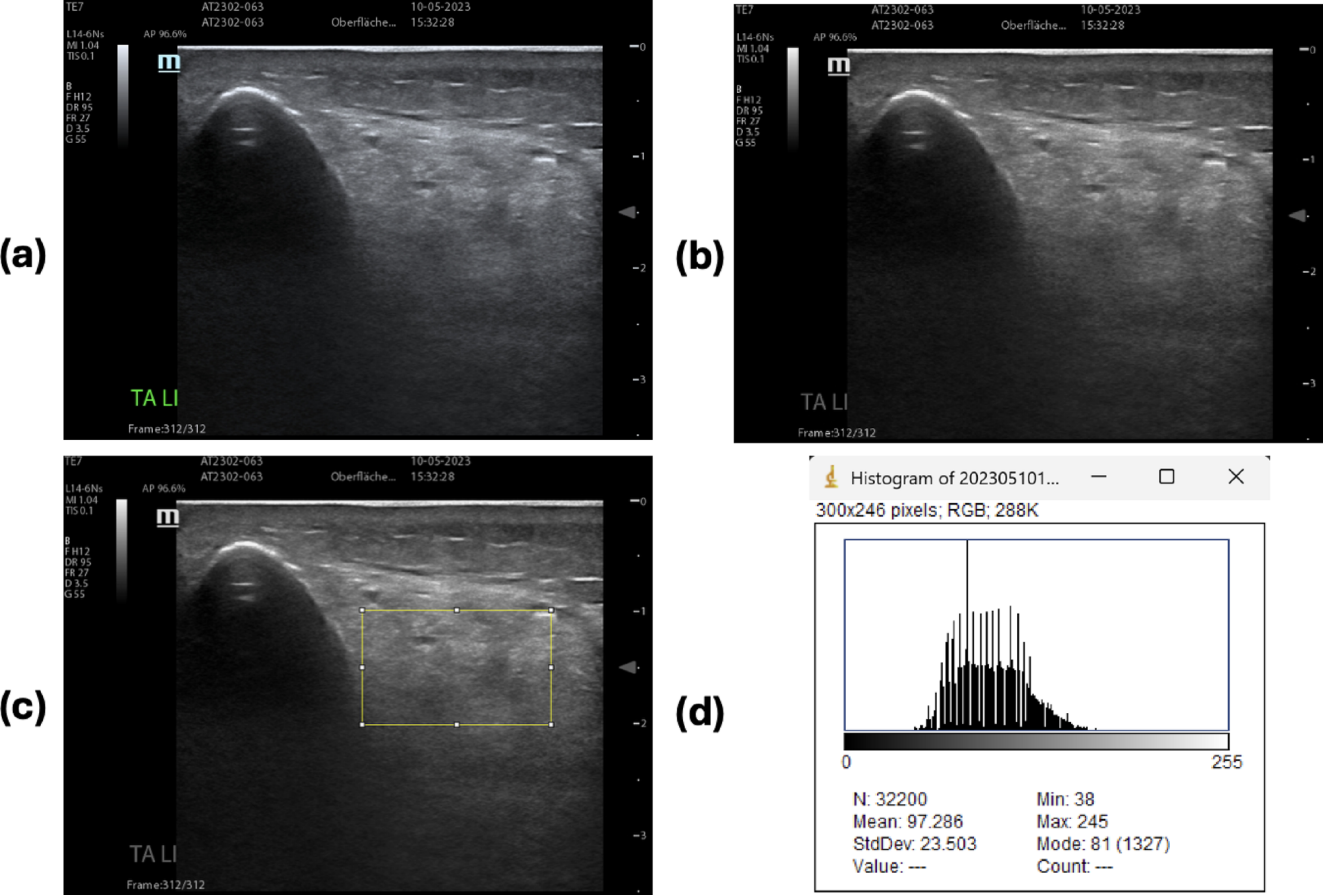
Sample images for grayscale analysis. (**a**) „Raw“ image of tibial anterior muscle (TA). The gain was kept constant throughout all examinations. The focus for this muscle was set at a depth of 1.5 cm. (**b**) Conversion to an 8-bit image. (**c**) Selection of the region of interest (ROI). For TA, it was selected at 0.5 cm above and below the focal point (corresponding to 140 pixels) with a standardized width of 240 pixels. (**d**) Corresponding Histogram analysis

In addition, the muscle echogenicity of the biceps brachialis (BB), brachioradialis (BR), first dorsal interosseous (FDI), tibialis anterior (TA), gastrocnemius lateralis (GCNL), and gastrocnemius medialis (GCNM) muscles was determined, each on the non-dominant side. Angle of insonation, gain and imaging setups were kept constant throughout the whole examination and between the individual examination points. Determination of muscle echogenicity was done with six patients at baseline and with twelve patients at follow-up. We used the Heckmatt grading scale for semiquantitative classification, ranging from 1 point for normal muscle to 4 points for muscles with maximum alteration [28]. For further analysis, muscle ultrasound images were converted to 8-bit grey value images. Grayscale histogram analysis was performed on these images using ImageJ software. To this end, ROIs were marked according to the ultrasound focal zone to cover as much muscle as possible (Fig. 2).

### Statistical analysis

All statistical analyses were performed using GraphPad Prism 8.0.2 for windows (Graphpad Software, San Diego, California, USA, www.graphpad.com). Data distribution was assessed with the Kolmogorov–Smirnov and Shapiro-Wilk tests. Normally distributed data were analyzed using paired *t*-tests for longitudinal comparisons; non-normally distributed data were analyzed using the Wilcoxon signed-rank test. Differences between two independent, non-normally distributed groups were evaluated using the Kolmogorov–Smirnov test for two samples. Associations between clinical scores (e.g., CMTESv2, MRC) and imaging parameters (e.g., nSI, PDFF, UPSS, CSA) were examined using linear regression models with calculation of the coefficient of determination (*r²*) and two-tailed *p*-values. Data are reported as mean ± standard deviation, unless stated otherwise. Statistical significance was defined as *p* < 0.05. Given the study‘s exploratory character and limited sample size, no formal correction for multiple testing was applied.

## Results

A total of 14 patients with genetically confirmed hereditary polyneuropathies were included in this study (Table 2). The diagnoses were distributed as follows: CMT1A (*n* = 5; 3 female, 2 male), CMT1X (*n* = 3; 1 female, 2 male), CMT1B (*n* = 2; 1 female, 1 male), CMT1C (*n* = 2; 2 female), CMT2 (*n* = 1; female), and hereditary myoneuropathy (*n* = 1; female). Thus, two patients with axonal polyneuropathy (CMT2, and hereditary myoneuropathy) and 12 with demyelinating polyneuropathy (CMT1A, CMT1X, CMT1B, and CMT1C) were included. The average follow-up interval for all patients was 4.75 ± 0.39 years. Due to the genetic component of the diseases, the average age of onset was very young for most cases. The earliest average onset was 9.0 ± 1.41 years for CMT1C and 11.6 ± 10.43 years for CMT1A. CMT1X followed this with 17.67 ± 8.74 years, CMT1B with 21.5 ± 19.09 years, and the diseases with only one examined patient each, CMT2 (48 years), and hereditary myoneuropathy (52 years). The disease duration differed significantly between the groups of axonal vs. demyelinating polyneuropathy (6.0 ± 0 vs. 38.17 ± 13.16 years). All patients in this study were of Caucasian descent.

**Table 2:** Demographic, clinical, and imaging characteristics of the study cohort

Variable	All patients *[n = 15]*	CMT1A *[n = 5]*	CMT1X *[n = 3]*	CMT1B *[n = 2]*	CMT1C *[n = 2]*	CMT2 *[n = 1]*	Hereditary myoneuropathy *[n = 1]*
*Affected gene*	N/A	*PMP22*	*GJB1*	*MPZ*	*LITAF*	*DCNT2*	*DNAJB5*
*Mutations*	N/A	*PMP22 Dup (het)*	*1. c.479 A > G; p.Tyr160Cys (hem)* *2. c.479 A > G; p.Tyr160Cys (hem)* 3. c.617dupT; p.(Ala207Glyfs*36)	*c.616G > T; p.Gly206Ter (het)*	*c.430G > A; p.Val144Met (het)*	*c.1159 C > G; p.Leu387Val(het)*	c.85_89dupTGGAG; p.Arg30Serfs*50 (het)
*Sex [f: m]*	9:5	3:2	1:2	1:1	2:0	1:0	1:0
*Age [years]*	53 ± 13.3 (32–74)	53.2 ± 12.36 (40–69)	51.33 ± 15.53 (34–64)	57 ± 24.04 (40–74)	48 ± 22.63 (32–64)	54	58
*Height [cm]*	171.07 ± 7.74 (154–183)	166.2 ± 9.42 (154–178)	170.33 ± 2.52 (168–173)	174.5 ± 12.02 (166–183)	174 ± 1.42 (173–175)	176	180
*Weight [kg]*	86.21 ± 19.22 (57–137)	73.6 ± 6.35 (68–84)	76.33 ± 16.86 (57–88)	95 ± 7.07 (90–100)	116 ± 29.7 (95–137)	95	93
*CMTESv2 (0–28)*	13.71 ± 5.7	17.8 ± 4.09	16.33 ± 4.16	12 ± 2.83	10.5 ± 3.54	5	4
*Foot dorsiflexion MRC scale (0–5) baseline*	3.44 ± 1.47	2.4 ± 1.52	3.5 ± 0.5	5 ± 0	5 ± 0	5	5
*Foot dorsiflexion MRC scale (0–5) follow-up*	3.61 ± 1.67	2.3 ± 1.92	3.5 ± 1.15	5 ± 0	5 ± 0	5	5
*Foot plantarflexion MRC scale (0–5) baseline*	4.53 ± 0.93	3.8 ± 1.3	4.83 ± 0.29	5 ± 0	5 ± 0	5	5
*Foot plantarflexion MRC scale (0–5) follow-up*	4.36 ± 1.2	3.5 ± 2.06	3 ± 0	5 ± 0	5 ± 0	5	5
*VL muscle nSI baseline*	0.4 ± 0,05	0.41 ± 0.07	0.36 ± 0.02	0.38 ± 0.02	0.43 ± 0.04	0.45	0.38
*VL muscle nSI follow-up*	0.41 ± 0.06	0.4 ± 0.06	0.36 ± 0.03	0.4 ± 0.01	0.5 ± 0.038	0.43	0.41
*RF muscle nSI baseline*	0.35 ± 0.04	0.34 ± 0.05	0.35 ± 0.05	0.33 ± 0.01	0.38 ± 0.07	0.37	0.3
*RF muscle nSI follow-up*	0.32 ± 0.03	0.31 ± 0.04	0.3 ± 0.03	0.34 ± 0.00	0.37 ± 0.01	0.3	0.32
*TA muscle nSI baseline*	0.38 ± 0.1	0.41 ± 0.08	0.44 ± 0.18	0.33 ± 0.02	0.35 ± 0.06	0.29	0.3
*GCNL muscle nSI baseline*	0.41 ± 0.07	0.44 ± 0.09	0.36 ± 0.07	0.43 ± 0.05	0.43 ± 0.02	0.38	0.38
*GCNM muscle nSI baseline*	0.52 ± 0.2	0.56 ± 0.21	0.57 ± 0.36	0.44 ± 0.09	0.49 ± 0.72	0.39	0.45
*TA muscle nSI follow-up*	0.41 ± 0.12	0.45 ± 0.11	0.45 ± 0.2	0.34 ± 0.002	0.45 ± 0.08	0.31	0.29
*GCNL muscle nSI follow-up*	0.46 ± 0.1	0.54 ± 0.14	0.42 ± 0.06	0.43 ± 0.05	0.43 ± 0.01	0.39	0.41
*GCNM muscle nSI follow-up*	0.56 ± 0.21	0.65 ± 0.25	0.59 ± 0.31	0.46 ± 0.14	0.55 ± 0.13	0.39	0.44
*TA muscle echogenicity*	80.55 ± 19.79 *	99.01 ± 12.68*	70.5 ± 7.21	64.55 ± 37.64	77.38 ± 19.5	85.15	70.59
*GCNL muscle echogenicity*	73.67 ± 21.39	86.37 ± 17.7	62 ± 17.34	73.62 ± 19.41	43.38 ± 9.96	94.51	85.09
*GCNM muscle echogenicity*	74.9 ± 11.52 *	72.38 ± 13.39*	71.22 ± 16.02	79.42 ± 9.35	69.8 ± 1.07	90.81	81.35

Values are given as mean ± standard deviation (range) unless otherwise indicated. CMTESv2 = Charcot-Marie-Tooth Examination Score version 2; MRC = Medical Research Council scale; RF = rectus femoris; VL = vastus lateralis; TA = tibialis anterior; GCNL/GCNM = gastrocnemius lateralis/medialis; nSI = normalized signal intensity; muscle echogenicity = grayscale histogram value. Muscle echogenicity is only indicated here at the time of follow-up. One dataset was missing for echogenicity in CMT1A (*)

### Clinical symptomatology and nerve conduction studies

At the most recent visit, the most common complaints included paresthesia (93%), disturbance of fine motor skills (86%), numbness (79%), gait instability (79%), and muscle weakness (79%). The prevalence of pes cavus and claw toes was found to be 86% among the patient population. The sensory evaluation revealed the absence of cold perception in the lower limbs among all patients, along with a reduced perception of touch (79%) and pinpricking (79%). Perception in the upper limbs was greater but also limited in some patients (limitation of cold perception: 57% of patients; limitation of pinprick sensitivity: 36%; limitation of touch perception: 29%). All patients demonstrated the ability to walk independently. However, 21% of patients reported using orthopedic walking aids, such as foot-lifter orthoses. The results of the motor testing indicated that 79% of patients were not able to walk on their heels, and 50% exhibited an inability to perform a tightrope walk. Furthermore, 43% of patients showed a steppage gait. The Romberg standing test was pathologically abnormal in 43% of patients. At follow-up the mean MRC sum score was 60.61 ± 9.94 points, with lower limb muscle groups being more affected than upper limb muscles.

**Fig. 3:**
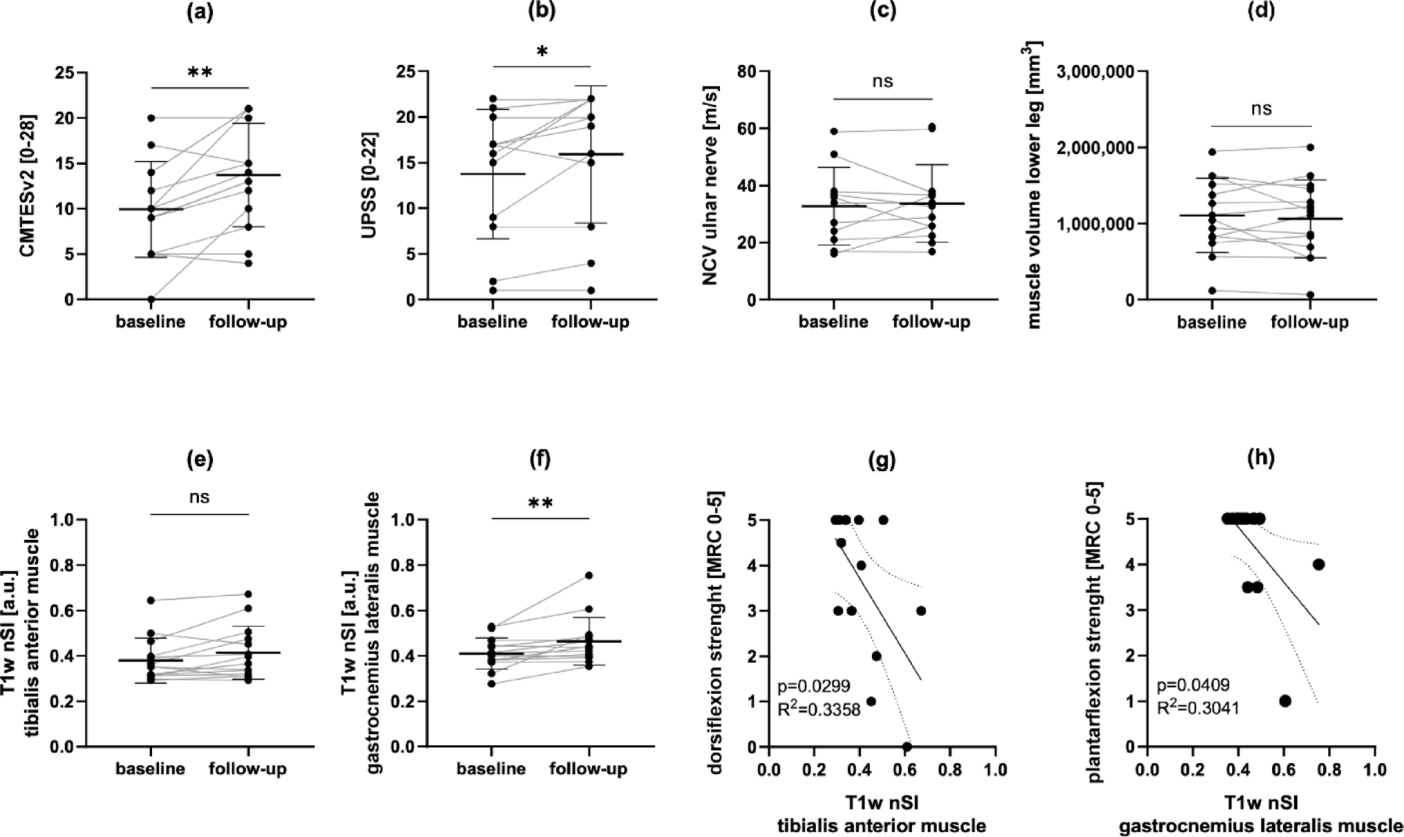
Longitudinal clinical, electrophysiological, MRI, and ultrasound findings in hereditary neuropathies. (**a**) Charcot-Marie-Tooth Examination Score version 2 (CMTESv2) significantly increased over the follow-up period, reflecting clinical progression. (**b**) The ultrasound pattern sum score (UPSS) increased in demyelinating neuropathies. (**c**) Ulnar nerve conduction velocity (NCV) showed no significant longitudinal change. (**d**) Lower leg muscle volume remained stable. (**e**–**f**) Normalized T1-weighted signal intensity (nSI) increased in the tibialis anterior (TA) and gastrocnemius lateralis (GCNL) muscles, indicating progressive fat infiltration in GCNL. (**g**–**h**) Foot dorsiflexion and plantarflexion strength (Medical Research Council, MRC scale) inversely correlated with nSI of tibialis anterior (TA) and gastrocnemius lateralis (GCNL) muscles. * = *p* < 0.05, ** = *p* < 0.01, ns = not significant

The mean CMT Examination Score version 2 (CMTESv2) demonstrated a significant increase from 9.93 ± 5.28 to 13.71 ± 5.7 over the course of the follow-up period, indicating progression in the clinical manifestation of symptoms (*p* = 0.0031; Fig. 3a). Progression was particularly evident in the patient’s motor symptoms (CMTESv2 motor categories: 3.29 ± 2.92 vs. 6.28 ± 3.6; *p* = 0.0012), whereas no significant increase was observed in the sensory items of the CMTESv2 (CMTESv2 sensory categories: 6.64 ± 3.08 vs. 7.43 ± 2.56; *p* = 0.18).

NCS showed slowed ulnar NCV in 85.7% of patients. Nearly all patients (92.9%) had reduced or absent radial SNAP. Patients showed no significant progression in NCS over the follow-up period (Fig. 3c).

### Muscle volumetry and MRI signal intensity

Quantitative volumetry of the lower limb muscles showed no significant changes over the follow-up period. The mean muscle volume of the thigh was 3,521 ± 908.53 cm³ at baseline and 3,488 ± 778.68 cm³ at follow-up. The mean muscle volume of the lower leg was 1,106 ± 487.69 cm³ at baseline and 1,061 ± 510.14 cm³ at follow-up (Fig. 3d).

**Fig. 4:**
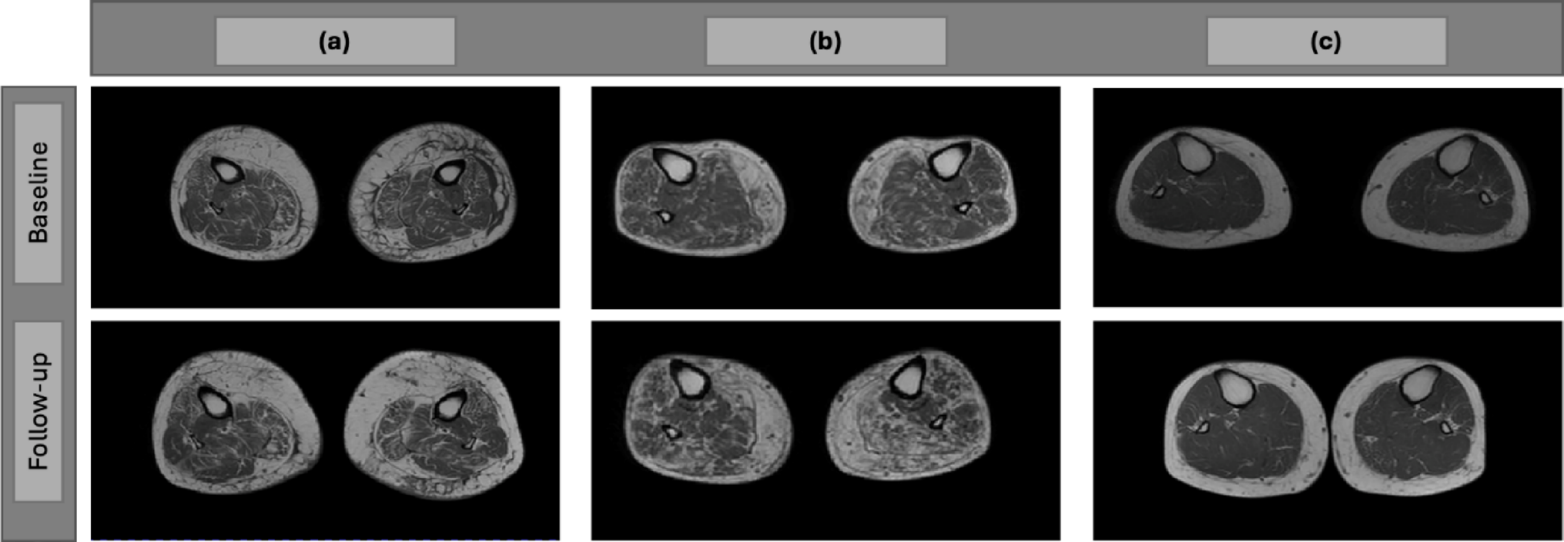
Longitudinal MR image examples of lower leg muscles in hereditary neuropathies. Representative axial T1-weighted MR images at baseline (top row) and follow-up (bottom row) in three patients: (**a**) 64-year-old female with CMT1C, (**b**) 69-year-old male with CMT1A, and (**c**) 58-year-old female with hereditary myoneuropathy. Progressive fatty infiltration of lower leg muscles is most evident in the patient with CMT1A (**b**), whereas changes are less pronounced in CMT1C (**a**) and hereditary myoneuropathy (**c**). Images illustrate the heterogeneous course of disease progression across different genotypes

Analysis of muscle nSI on MRI showed a mild increase over time in the GCNL muscle. In contrast, GCNM, TA and proximal muscles showed no significant change. The mean nSI of distal leg muscles increased from 0.44 ± 0.14 at baseline to 0.48 ± 0.16 at follow-up compared to 0.373 ± 0.05 at baseline and 0.366 ± 0.07 at follow-up. Region-specific analysis on the distal leg muscles showed that at follow-up, the GCNM had the highest nSI value with 0.56 ± 0.21, followed by the GCNL with 0.46 ± 0.1 and the TA with 0.41 ± 0.12. In comparison proximal leg muscles showed slightly lower nSI values at follow-up. The follow-up nSI of the RF muscle was 0.32 ± 0.03 and of the VL muscle 0.41 ± 0.06. Longitudinal analysis showed a significant increase in nSI in the GCNL (0.41 ± 0.07 vs. 0.46 ± 0.1, *p* = 0.008; Fig. 3f). In contrast, no significant change was observed in the TA (*p* = 0.052) and GCNM (*p* = 0.08) as well as the proximal muscles (VL: *p* = 0.160; RF: *p* = 0.1154). In addition to the partially visible change in muscle composition (Fig. 4), the increase in nSI indicates an increase in the fat content of the muscle. The lack of change in the GCNM could be explained by the fact that this muscle was already most severely affected at baseline, leaving less room for deterioration.

Comparison of the nSI of the TA and foot dorsiflexion strength revealed an inverse correlation (*p* = 0.03, r² = 0.34, Fig. 3g). Similarly, comparisons between foot plantarflexion strength and the nSI of the GCNM and GCNL revealed an inverse correlation (GCNM: *p* = 0.047, r² = 0.29; GCNL: *p* = 0.041, r² = 0.3, Fig. 3h).

### Ultrasound findings

High-resolution muscle ultrasound (HRUS) revealed the highest mean muscle echogenicity in the TA muscle (80.55 ± 19.79) at the second examination. This was followed by the GCNM (74.9 ± 11.52) and GCNL (73.67 ± 21.39) muscles. Muscles of the upper extremities showed lower muscle echogenicity values: FDI muscle (47,57 ± 22.2), BR muscle (64.24 ± 21.46), BB muscle (68.02 ± 23.78).

Muscle echogenicity in the FDI muscle significantly correlated with the CMAP of the ulnar nerve (*p* = 0.0283, r^2^ = 0.4305). However, no significant correlation was detected between muscle echogenicity and corresponding MRI signal intensities.

The UPSS appeared lower in the axonal cohort compared to the demyelinating cohort; however, no statistically significant difference was observed, likely due to the very small sample size of the axonal group (*n* = 2, 4 ± 4.24 points vs. *n* = 12, 15.92 ± 7.5 points). Longitudinal analysis showed that the demyelinating cohort showed a significant increase in UPSS (13.75 ± 7.1 vs. 15.92 ± 7.5; *p* = 0.03; Fig. 3b). The CMT1B group had the highest mean UPSS (22 ± 0), followed by the CMT1A group (20.6 ± 1.34).

Analysis of nerve CSA and correlation with findings of MRI nSI showed no correlation, e.g., CSA of the peroneus nerve showed no correlation with the nSI of the TA (*p* = 0.69, r^2^ = 0.013). No significant correlation between CSA and nerve conduction velocity was found in our cohort. The UPSS showed an inverse correlation with the NCV of both the median nerve (r^2^ = 0.42, *p* = 0.04) and the ulnar nerve (r^2^ = 0.51, *p* = 0.006).

## Discussion

Our longitudinal study provides a comprehensive evaluation of disease progression in patients with genetically confirmed hereditary polyneuropathies over an average follow-up period of 4.75 years. The increase in the CMTESv2 Score, especially related to motor symptoms, confirms the progressive nature of hereditary polyneuropathies.

This study focused on lower limb muscle involvement, in line with numerous previous MRI studies that primarily assessed proximal and distal muscles of the lower extremities [29–31]. In comparison, proximal muscles showed lower nSI than lower leg muscles which is in line with findings of other studies, stating lower legs muscles being most affected by fatty replacements in hereditary polyneuropathies [32, 33].

Previous studies have demonstrated that muscle MRI provides valuable diagnostic information and can help differentiate between various neuromuscular disorders, including hereditary and acquired neuropathies, based on distinct patterns of muscle involvement. For example, characteristic distributions of fatty infiltration and selective muscle involvement have been described in different genetic and clinical subtypes, supporting the role of MRI as a non-invasive diagnostic tool [31, 34, 35]. However, the primary aim of our study was not to differentiate between neuropathy subtypes. Instead, we focused on the longitudinal assessment of disease progression using muscle MRI in combination with high-resolution ultrasound (HRUS) in hereditary neuropathies.

As the most important result, MRI showed a slight change in muscle composition, indicated by the increase of nSI, whereas HRUS was not sensitive to clinical progression. In line with these MRI changes, especially the patients’ motor symptoms have progressed over the follow-up time. We therefore conclude that measuring PDFF or nSI as its surrogate parameter is a sensitive way of recognizing changes and disease progression, this can be particularly interesting when the disease has not yet reached an advanced stage. Of note, mDIXON Quant or similar sequences for PDFF measurement may not be available at all MRI scanners, whereas T1w TSE sequences for nSI calculation are standard sequences. Kim et al. previously assessed mean normalized signal intensity derived from T1w lower leg MRIs in CMT1A patients as a potential imaging biomarker and demonstrated correlations with several clinical parameters [30] Although our study employed a slightly different methodological approach to determine the nSI, we were also able to demonstrate similar correlation between clinical outcome and nSI. In particular, we determined mean SIs in representative cylindrical ROIs and referenced them to the fatty bone marrow only, whereas Kim et al. used all segmented muscle and fatty tissues within a slice, respectively. This choice was made because of the presence of edema within the subcutaneous fat of some patients, which would have falsified our nSI calculations. In addition to Kim et al., we directly compared PDFF and nSI during follow-up. This comparison revealed a strong correlation between the two values, which was then used as an internal validation for the use of nSI as a surrogate marker within our cohort.

Quantitative evaluation of muscle volumes showed no change. This indicates that this method was not precise enough to detect changes over a short period of time (i.e., over 4 years). Additionally, semi-automated segmentation proved to be error-prone. The more we had to improve the segmentation, the less reliable the results became. This could also be reflected in the slight, though not significant, increase in the thigh muscle volume over time. It is highly probable that this result was caused by measurement inaccuracies. Nevertheless, we consider semi-automated segmentation to be a useful tool for identifying initial atrophy patterns as shown by Bähr et al. [8].

In contrast to other studies, our ultrasound results showed no significant correlation between CSA and nerve conduction velocity, which might be explained by our small cohort size [36]. On the other hand, there was a significant correlation between the UPSS and NCV of the median and ulnar nerve. Our findings suggest that the NCV is not an accurate reflection of disease progression, as it showed no change over the follow-up period despite progression of clinical symptoms [37, 38]. The longitudinal evaluation showed an increase in UPSS only in demyelinating polyneuropathies. This has not been described in previous studies, which instead reported a constant CSA size in CMT1A patients [39]. Whether the UPSS can therefore be used as a disease progression marker needs to be further evaluated in larger prospective studies. We recommend validating this observation with a larger cohort to obtain more reliable results.

In our study, muscle echogenicity of the first dorsal interosseous muscle correlated with the ulnar CMAP amplitude, which we considered clinically meaningful. Previously published data from our own group [15] and others [40] suggest that in a larger cohort, more relevant correlations between muscle echogenicity and clinical data could be observed. Although muscle ultrasound is very well established in the diagnosis of neuromuscular diseases, there are some disadvantages in the assessment of disease progression: the echogenicity of the images is strongly dependent on the gain, the experience of the examiner and the system settings of the ultrasound device. This underlines the importance of standardized ultrasound acquisition [41]. Despite the same setup settings, an image comparison between two ultrasound devices is only possible with great effort using standardization curves and even then, only to a limited extent. Determination of the muscle diameter as a possible follow-up parameter is also limited or not possible in the case of hyperechogenic muscles (Heckmatt score ≥3). The same applies to deeper muscle groups. Muscle ultrasound can therefore only be used under specific conditions as a disease progression assessment.

Despite PDFF being an accurate and objective progression marker in this and other cohorts, MRI is a costly examination that might not be available for all neuropathy patients in clinical practice. HRUS is another valuable addition in this respect, being much easier and faster to obtain. Additionally, patients generally find the examination much more pleasant than nerve conduction studies. It is a good marker for diagnosing and differentiating hereditary polyneuropathy, particularly when using the UPSS survey. In case of demyelinating polyneuropathies, it could also indicate disease progression.

This study’s small sample size and heterogenous population is a limitation. This is an inherent challenge in the study of rare diseases. Unlike other studies, the present analysis includes patients treated in routine clinical practice. While this inevitably results in increased heterogeneity, it more accurately reflects everyday clinical care. Further studies in larger genotype-specific cohorts are needed to validate the biomarkers investigated in this study. Due to unreliable semi-automatic segmentation, manual improvement was often necessary. This may have resulted in more operator-dependent segmentation. Additionally, minor inaccuracies may have influenced the results of the CSA determination by ultrasound, as a potential measurement bias cannot be fully excluded. Due to a protocol change in the early stage of this study, baseline muscle echogenicity data was incomplete. This limited our ability to conduct a longitudinal analysis of muscle echogenicity, including the Heckmatt score and grayscale analysis. An important limitation is that disease duration was not included as a covariate in the regression analyses. Given the strong influence of cumulative disease burden on muscle degeneration in hereditary neuropathies, the observed regional variations in nSI could potentially reflect variations in disease duration among patients as well as region- and pathology-specific vulnerability.

The study’s inclusion of MRI and HRUS data is a particular strength. Unfortunately, our study lacks longitudinal PDFF data. However, as such data were available at the second time point only, we used it for internal validation of our normalized nSI as a surrogate biomarker for PDFF. The reader should be aware that nSI can be subject to influences other than the fat content. For example, the sequence parameters of the T1w sequence will influence the brightness of different tissues. Our calibration curve may therefore not be transferable to data from other centers. As in the work by Kim et al.^30^, we showed correlations with clinical outcome parameters.

A major strength of this study is its longitudinal design. Such data are difficult to obtain, particularly in the field of rare diseases such as hereditary polyneuropathies. Our cohort represents a real-world clinical patient cohort and, thus, provides results with a more practical orientation.

## Conclusion

This exploratory longitudinal study demonstrates that MRI, particularly MRI-derived quantitative parameters, such as PDFF and nSI, provide valid biomarkers of structural progression in hereditary neuropathies. In contrast, muscle volumetry and NCS lacked responsiveness to clinical changes over a five-year interval. HRUS, especially the ultrasound pattern sum score UPSS, showed potential for monitoring disease progression in demyelinating but not axonal subtypes. Overall, MRI and HRUS should be used as complementary imaging modalities in hereditary neuropathies. Their application may improve the sensitivity of outcome measures in forthcoming clinical trials and aid in evaluating emerging therapies. Validation of these biomarkers in larger, genotype-specific cohorts will be essential to establish their role in routine clinical practice.

## Data Availability

The data are available from the corresponding author on reasonable request.

## Abbreviations

BB: Biceps brachii muscle
BR: Brachioradialis muscle
CMAP: Compound motor action potential amplitudes
CMT: Charcot-Marie-Tooth disease
CMTESv2: Charcot-Marie-Tooth examination score version 2
CSA: Nerve cross-sectional area
FDI: First dorsal interosseous muscle
DML: Distal motor latencies
GCNL: Gastrocnemius lateralis muscle
GCNM: Gastrocnemius medialis muscle
HRUS: High-resolution ultrasound
mDIXON: Modified dixon
MRC: Medical research council scale
MRI: Magnetic resonance imaging
NCS: Nerve conduction studies
NCV: Nerve conduction velocities
nSI: Normalized T1w signal intensity
PDFF: Proton density fat fraction
RF: Rectus femoris muscle
ROI: Region of interest
SI T1w: Signal intensity
SNAP: Sensory action potential
STIR: Short tau inversion recovery
T1w: T1-weighted
TA: Tibial anterior muscle
TSE: Turbo spin echo
UPSS: Ultrasound pattern sum score
VL: Vastus lateralis muscle
